# Ketogenic Diet in Super-Refractory Status Epilepticus

**DOI:** 10.15844/pedneurbriefs-31-3-2

**Published:** 2017-11

**Authors:** Garnett Smith, Craig A. Press

**Affiliations:** 1 Department of Pediatrics, Section of Neurology, School of Medicine, University of Colorado Anschutz Medical Campus, Aurora, CO

**Keywords:** Ketogenic Diet, Super Refractory Status Epilepticus, Pediatrics, Epilepsy

## Abstract

Researchers from the Children’s National Health System in Washington, D.C. studied the feasibility, rate of complications, and effect on seizures of initiating the Ketogenic Diet (KD) in pediatric patients with Super-Refractory Status Epilepticus (SRSE).

Researchers from the Children’s National Health System in Washington, D.C. studied the feasibility, rate of complications, and effect on seizures of initiating the Ketogenic Diet (KD) in pediatric patients with Super-Refractory Status Epilepticus (SRSE). They retrospectively identified nine children, presenting between 2009 and 2016, for whom KD was initiated in the pediatric intensive care unit to treat SRSE. The median time to initiation of KD was 13 days [interquartile range (IQR) 10 – 16]. By the time of initiation of KD, the median number of seizure medications trialed was 4 [IQR 3-4] and the median number of continuous infusions received prior to initiation of KD was 2 [IQR 2-3]. After initiation of KD, the mean time to achieve ketosis was 4.2 days [SD 3.4] and most patients were weaned off continuous infusions of anesthetics by one week. Three patients experienced hypoglycemic events during the first week of KD therapy. Two patients developed severe hypertriglyceridemia, both of whom were on intravenous KD. Four patients were seizure free within one week of initiation of KD, with one patient showing no improvement in seizure frequency at one week. At three months, seizure burden and functional outcome were variable, with most continuing to experience seizures and require assistance with activities of daily living. [1]

COMMENTARY. SRSE has been defined as status epilepticus that continues 24 hours after initiation of general anesthesia, including cases where status epilepticus recurs after reduction of anesthesia [2]. Prior reports demonstrate that outcomes in SRSE are very poor, with hospital mortality ranging from 30% – 40%, and about 75% of patients experiencing a poor functional outcome at discharge [3, 4]. There are no randomized controlled trials for treatment of SRSE. A retrospective study of 10 adults treated with KD for SRSE demonstrated similar findings with 9/10 achieving ketosis in a median of 3 days. Seven of 10 had resolution of SRSE [5]. One limitation of these studies is their retrospective nature without a standard protocol or criteria for weaning anesthesia or determining the resolution of SRSE. It is not uncommon to tolerate some frequency of seizure during anesthetic weans when they have failed in the past. Additionally, it is unclear if additional therapies were added in proximity to the initiation of KD which complicates the determination of the effectiveness of KD.

Initiation of KD in the ICU setting is associated with significant challenges including: multiple intravenous infusions, difficulty eliminating all sources of carbohydrate, use of corticosteroids which can inhibit the establishment of ketosis, and inability to provide enteral nutrition. Although generally well-tolerated, KD can also cause complications including hypoglycemia, hepatotoxicity, cholestasis, or hypertriglyceridemia. A consistent and thoughtful approach allowed for initiation of KD while monitoring for complications.

The current study adds to available knowledge about KD in SRSE by contributing additional evidence of the feasibility and safety of KD in this setting. It also provides an overview of the protocol that the authors use in this situation. Further study is necessary to understand outcomes of seizure frequency and functional status when KD is used to treat SRSE.
